# Application of principal component analysis for the optimisation of lead(II) biosorption

**DOI:** 10.1007/s11274-017-2358-7

**Published:** 2017-10-03

**Authors:** Łukasz Wajda, Aleksandra Duda-Chodak, Tomasz Tarko, Paweł Kamiński

**Affiliations:** 1 Malopolska Centre of Biotechnology, Jagiellonian University, Gronostajowa 7A Str, 30-387 Krakow, Poland; 2 Department of Fermentation Technology and Technical Microbiology, Faculty of Food Technology, University of Agriculture in Krakow, ul. Balicka 122, 30-149 Krakow, Poland; 3 Department of Geomechanics, Civil Engineering and Geotechnics, AGH University of Science and Technology, al. Mickiewicza 30, 30-059 Krakow, Poland

**Keywords:** *Arthrospira platensis*, Lead, Paper filter, Biosorption, Principal component analysis

## Abstract

Current study was focused on optimising lead(II) biosorption carried out by living cells of *Arthrospira platensis* using Principal Component Analysis. Various experimental conditions were considered: initial metal concentration (50 and 100 mg/l), solution pH (4.0, 4.5, 5.0, 5.5) and contact time (10, 20, 30, 40, 50 and 60 min) at constant rotary speed 200 rpm. It was found that when the biomass was separated from experimental solutions by the filtration, almost 50% of initial metal dose was removed by the filter paper. Moreover, pH was the most important parameter influencing examined processes. The Principal Component Analysis indicated that the most optimum conditions for lead(II) biosorption were metal initial concentration 100 mg/l, pH 4.5 and time 60 min. According to the analysis of the first component it might be stated that the lead(II) uptake increases in time. In overall, it was found to be useful for analysing data obtained in biosorption experiments and eliminating insignificant experimental conditions. Experimental data fitted Langmuir and Dubinin–Radushkevich models indicating that physical and chemical absorption take place at the same time. Further studies are necessary to verify how sorption–desorption cycles affect *A. platensis* cells.

## Introduction

Lead is toxic at very low doses and it accumulates in tissues of living organisms. Moreover, this element is the only heavy metal which does not pose any beneficial effects to human body (Damstra 1977). Currently, except from workers employed in industries involving heavy metal processing, children are mostly subjected to lead exposure (Damstra 1977). Therefore, it is necessary to prevent contamination of potable water or food with that element. The group of alternative technologies applied for gaining that goal are based on biosorption—processes involving living or inactivated biomass for heavy metal recovery (Volesky and Naja 2005).

Cyanobacteria belonging to the *Arthrospira* genus have been tested as biosorbents in various studies (Augusto Da Costa and De França 1998; Gong et al. 2005a; Chen and Pan 2005a, b; Vannela and Verma 2006; Gokhale et al. 2008; Lodi et al. 2008). According to Chen and Pan (2005a) who carried out the research involving living cells of *Arthrospira platensis*, the biomass removed lead from solutions at concentrations below 10 mg/l. Moreover, in our previous study (Duda-Chodak et al. 2013), we successfully conducted biosorption at higher lead concentrations by engaging immobilised cyanobacterium biomass and at the same time we demonstrated that free cells of *Arthrospira platensis* survived for at least 24 h in solutions containing up to 100 mg Pb/l regardless the pH of solutions. Therefore, we decided to continue the research involving living cells of *A. platensis* for lead biosorption. We applied Principal Component Analysis (PCA) for analysing that phenomenon and for selecting most optimum parameters of that process.

PCA has been scarcely used for describing biosorption experiments. It has been already applied for optimising bioremediation of dyes used in the food industry with Pittsburgh commercial activated carbon (Al-Degs et al. 2012). It also allowed formulating mathematical model describing those processes. Moreover, the PCA has been applied for optimising the removal of orange 12 dye by activated carbon coated with copper sulfide nanoparticles (Ghaedi et al. 2014). Another research which involved PCA was focusing on soil bioremediation with indigenous microflora but PCA was only used for interpreting patterns obtained by denaturing gradient gel electrophoresis (DGGE), not for describing biosorption processes (Chen et al. 2011). PCA also proved to be very useful tool for analysing metal removal by two species of earthworm and it allowed comparing DNA sequences of those organisms (Dai et al. 2004). It also indicated how each metal influenced Biota-to-Soil accumulation factors.

However, PCA has not been used for optimising heavy metal biosorption so, to the best of our knowledge, current study is the first where that statistical tool was applied. We determined optimum pH, lead initial concentration and contact time of the biosorbent with the solution. Moreover, we discovered that lead is absorbed by filter paper used for separating the cyanobacterium biomass from model solutions.

## Materials and methods

If not stated otherwise, all chemicals used in the study were manufactured by POCh (Gliwice, Poland). *Arthrospira platensis* (SAG 257.80) was purchased from Sammlung von Algenkulturen Universität Göttingen and cultivated in Zarrouk medium.

### Cyanobacterium culture

The biomass was cultivated at 20 ± 1 °C under a fluorescent lamp 40 W, 2000–3000 lx in cycles of 12 h of light followed by 12 h of darkness. Cell growth was determined by determining dry matter content (laboratory dryer, 105 °C, 2 h) which was sufficient for further experiments after approximately 3–4 weeks. Cells were collected by centrifugation (2750*g*, 20 °C), washed thoroughly with deionized water and centrifuged again under the same conditions. The cell pellet was re-suspended in 25 ml of deionized water and will be further referred to as the ‘cell suspension’. The dry matter content of cell suspension was determined each time using a moisture analyser (MAC50, Radwag, Poland).

### Biosorption experiments

Lead solutions (50 or 100 mg Pb^2+^/L) were prepared using analytical grade lead nitrate [Pb(NO_3_)_2_] and their pH (4.0, 4.5, 5.0, and 5.5) was adjusted with 0.1 M NaOH or 0.1 M HCl. All glassware was washed with 3% HNO_3_ (12 h) before and after each experiment to wash out all potential contaminants. Cell suspension (1.000 ± 0.001 g) was introduced into 50 ml of lead solution containing different initial metal dose (50 or 100 mg Pb/L) and holding various initial pH (4.0, 4.5, 5.0, and 5.5). Then samples were incubated with continuous shaking (200 rpm) at room temperature. Cyanobacterium cells were removed from solutions by filtration (filter paper, grade 3 m/N, diameter 110 mm, Munktell, Sweden) after 10, 20, 30, 40, 50, or 60 min.

Obtained filtrates were adjusted to pH 2 with 1 M nitric(V) acid and lead concentration was determined by atomic absorption spectrophotometry (AAS) (Varian AA 240 FS, Varian Inc. Agilent Technologies). In the case of control samples (lead solutions without cell suspension) filter paper disks were mineralized after estimated time with concentrated nitric(V) acid (MARSXpress Microwave Digestion System, Warszawa Poland) at 170 °C for 15 min. The quantity of metal absorbed by the filter was determined as in the case of liquid samples. All experiments were performed at least in three replicates.

### Sorption isotherms

The lead uptake (*q*) of cyanobacterium biomass was calculated twice. Firstly, the following mass balance equation (Eq. 1) for the biosorbent was used (Volesky 2004):

1 $$q={\text{~}}\frac{{\left[ {V \times \left( {{C_0} - ~{C_f}} \right)} \right]}}{S}$$

For the second time, the corrected equation (Eq. 2) which considers the quantity of lead absorbed by the filter paper was used:

2 $${q_{corr}}=\frac{{\left[ {V \times \left( {{C_i} - ~{C_f}} \right)} \right]}}{S}$$

In both equations, the following symbols were used: *q—*lead uptake at equilibrium (mg Pb/g biosorbent dry matter); *V*—volume of metal-bearing solution (l); *C*_*0*_ *—*initial lead concentration (mg/l); *Ci—*corrected lead concentration after subtracting the quantity of the metal absorbed by the paper filter (mg/l); *C*_*f*_ —final lead concentration (mg/l); *S*—dry matter of the biosorbent (g).

Langmuir isotherm was based on the following equation (Eq. 3):

3 $${q_{corr}}=~\frac{{({Q_{max}}~ \times b \times {C_i})}}{{(1+b~ \times ~{C_i})}}$$

*Q*_*max*_ and *b* are Langmuir constants indicating maximum sorption capacities (mg/mg of dry matter) and sorbent/sorbate affinity, respectively (Volesky 2004) while *q*_*e*_ is the amount of metal adsorbed at equilibrium. Both constants were calculated by plotting *1*/*q*_*corr*_ versus *1*/*C*_*i*_ and adding trend line. Then *Q*_*max*_ and *b* were obtained as follows: *slope* = *1*/*(Q*_*max*_ *·b)* and *intercept* = *1*/*b*. Langmuir isotherm was obtained by plotting *C*_*i*_/*q*_*corr*_ versus *C*_*i*_.

Linearised form of Freundlich model applied in the current study was as follows (Eq. 4):

4 $${q_{corr}}=k~ \times ~C_{i}^{{1/n}}$$

where *k* and *n* are Freundlich constants were obtained by plotting *log(q*_*corr*_ *)* versus *log(C*_*i*_ *)* while *q*_*e*_ is the amount of metal adsorbed at equilibrium. Constants were calculated according to Volesky (2004).

Another biosorption isotherm which was examined in the current study was Dubinin–Radushkevich isotherm (Dada et al. 2012) (Eq. 5):

5 $$\ln {q_{corr}}=\ln {q_s} - ~{K_{ad}} \times ~{\varepsilon ^2}$$

where q_s_ is the theoretical isotherm saturation capacity (mg Pb^2+^/g), K_ad_ is Dubinin–Radushkevich isotherm constant (mol^2^/kJ^2^). Mean free energy of metal ions (E) was obtained from the Eq. (6) following equation where B_DR_ is the isotherm constant:

6 $$E=~\frac{1}{{\sqrt {2~ \times ~{B_{DR}}} }}$$

While ε constant was obtained in Eq. (7):

7 $$\varepsilon =RT\left( {1+~\frac{1}{{{C_f}}}} \right)$$

R is the gas constant (8.314 J/mol K) and T is the absolute temperature (301.15 K). Isotherm parameters were calculated from the slope (K_ad_) of the plot of lnq_cor_ versus ε^2^ and the exponent calculated from the intercept of the plot gave q_m_ (Erhayem et al. 2015).

Lagergren pseudo-first (Eq. 8) and pseudo-second (Eq. 9) order equations were applied for estimating kinetic models as in Erhayem et al. (2015):

8 $$\log \left( {{q_e} - ~{q_t}} \right)=\log {q_e} - ~\left( {\frac{{{k_1}}}{{2.303}}} \right)t$$

9 $$\frac{t}{{{q_t}}}=~\frac{1}{{{k_2}q_{2}^{2}}}+~\frac{t}{{{q_2}}}~t$$

Symbols used in the equations demonstrated above were: q_e_—the adsorption equilibrium capacity (mg/g), q_t_—the quantity of lead(II) adsorbed (mg/g) at time t, k_1_—the rate constant of pseudo-first-order (min ^−1^), q_2_—the maximum adsorption capacity (mg/g) and k_2_—the rate constant of pseudo-second-order (g/mg·min). Constants (k_1_, k_2_ and q_2_) were obtained from slope and intercept of pseudo-first-order and pseudo-second-order plots between log(q_e_ – q_t_) versus t and t/q_t_ versus t.

### Statistical analysis

All experiments were carried out at least in triplicates. The results are shown as arithmetic means (± standard deviation). The normality of distribution was assessed by Shapiro–Wilk test and significance of differences between means was assessed by one-way variance analysis (ANOVA) with post hoc Tukey test. The Principal Component Analysis (PCA) with varimax rotation was applied to assess correlations among variables. All statistical analyses were carried out using R: A language and environment for statistical computing, version 3.1.3 (Foundation for Statistical Computing, Vienna, Austria, 2015). ANOVA was carried out using “lm” function and Tukey test was done using “HSD.test” function in the “agricolae” package. The PCA was carried out in “psych” package (Beaumont 2012). Cortest-Bartlett test was carried out using “cortest.bartlett” function in “psych” package as well. The data demonstrated normal distribution so its transformation was not necessary. Strong correlations between loads and scores were considered when values obtained in correlation matrix exceeded 0.3.

## Results

It seems that pH 4.0 was optimum when lead initial concentration 50 mg/l, while pH 4.5 supported metal recovery at 100 mg/l (Tables 1, 2). Equilibrium was reached after 40 min at most of cases. At both initial lead concentrations, the quantity of metal adsorbed by paper disks did not exceed half of its initial quantity in experimental solutions (Tables 1, 2). Due to that fact, all q values were recalculated according to the Eq. (2) and it appears that the efficiency of lead removal did not exceed 70%, while it was approximately 90% before implementing corrections to the Eq. (1) (Tables 1, 2).

**Table 1:** The comparison of lead uptake (q) by *A. platensis* at Pb^2+^ initial concentration 50 mg/l calculated per Eqs. (1, 2)

pH	Contact time [min]	Corrected lead concentration C_i_ [mg/l]	Final lead concentration C_f_ [mg/l]	Lead uptake q (Eq. 1) [mg/g d.m.]	Corrected lead uptake q_corr_ (Eq. 2) [mg/g d.m.]
4.0	10	26.76	14.30 ± 1.43	54.09 ± 1.94^b^	18.9 ± 4.1^b^
	20		9.62 ± 0.96	61.19 ± 1.32^b^	26.0 ± 6.7^b^
	30		6.93 ± 0.69	65.26 ± 0.92^b^	30.0 ± 6.5^b^
	40		9.52 ± 0.95	134.93 ± 2.88^a^	57.5 ± 4.9^a^
	50		10.56 ± 1.06	131.46 ± 3.11^a^	54.0 ± 8.8^a^
	60		10.67 ± 1.07	131.10 ± 3.13^a^	53.6 ± 3.3^c,d^
4.5	10	18.52	16.53 ± 1.65	79.69 ± 3.52^c,d^	4.7 ± 2.4^d^
	20		17.94 ± 1.79	76.33 ± 3.23^d^	1.4 ± 0.0^d^
	30		16.46 ± 1.65	79.86 ± 3.51^b,c,d^	4.9 ± 3.6^b,c,d^
	40		12.86 ± 1.29	88.43 ± 2.80^a^	13.5 ± 1.6^a^
	50		14.71 ± 1.47	84.02 ± 3.09^a,b,c^	9.1 ± 1.6^a,b.c^
	60		13.50 ± 1.35	86.90 ± 2.82^a,b^	11.9 ± 3.8^a,b^
5.0	10	21.12	7.36 ± 0.74	152.29 ± 2.40^a^	49.1 ± 1.1^a^
	20		19.80 ± 1.98	107.85 ± 6.39^b^	7.3 ± 3.5^b^
	30		19.94 ± 1.99	107.36 ± 6.39^b^	6.3 ± 4.3^b^
	40		14.75 ± 1.47	125.89 ± 4.63^b^	22.8 ± 3.5^b^
	50		19.19 ± 1.92	110.04 ± 6.23^b^	6.9 ± 5.4^b^
	60		17.69 ± 1.77	115.39 ± 5.59^b^	12.2 ± 5.4^b^
5.5	10	14.03	12.90 ± 1.29	92.75 ± 2.91^b^	2.8 ± 0.0^b^
	20		4.35 ± 0.43	114.13 ± 0.92^a^	24.2 ± 1.2^a^
	30		5.94 ± 0.59	110.15 ± 1.23^a^	20.3 ± 0.9^a^
	40		7.73 ± 0.77	105.68 ± 1.79^a^	15.8 ± 1.4^a^
	50		9.06 ± 0.91	102.35 ± 1.90^a,b^	12.4 ± 7.0^a,b^
	60		8.88 ± 0.89	102.80 ± 2.01^a,b^	12.9 ± 3.3^a,b^

^a,b,c^The same letters next to values of lead uptake calculated according to the Eq. (1) or Eq. (2) at certain pH value (column) indicate the lack of statistically significant differences between means (p < 0.05), n = 6

**Table 2:** The comparison of lead uptake (q) by *A. platensis* at Pb^2+^ initial concentration 100 mg/l calculated per Eqs. (1, 2)

pH	Contact time [min]	Corrected lead concentration C_i_ [mg/l]	Final lead concentration C_f_ [mg/l]	Lead uptake q (Eq. 1) [mg/g d.m.]	Corrected lead uptake q_corr_ (Eq. 2) [mg/g d.m.]
4.0	10	68.38	35.42 ± 3.54	111.35 ± 5.40^c^	56.8 ± 6.4^c^
	20		33. 89 ± 3.39	113.98 ± 5.25^c^	59.5 ± 13.3^c^
	30		31.10 ± 3.11	118.79 ± 4.98^b.c^	64.3 ± 6.1^b,c^
	40		23.58 ± 2.36	131.76 ± 2.56^a^	77.2 ± 4.1^a^
	50		21.90 ± 2.19	134.66 ± 2.59^a^	80.1 ± 4.2^a^
	60		25.38 ± 2.54	128.66 ± 3.46 ^a,b^	74.1 ± 2.5^a,b^
4.5	10	64.77	34.14 ± 3.41	173.16 ± 5.72^d^	80.6 ± 12.4^d^
	20		29.53 ± 2.95	185.45 ± 3.59^b,c^	92.7 ± 9.8^b,c^
	30		29.33 ± 2.93	185.97 ± 5.49^b,c^	93.3 ± 9.9^b,c^
	40		31.33 ± 3.13	180.71 ± 6.06^c,d^	88.0 ± 9.2^b,c,d^
	50		26.15 ± 2.61	194.34 ± 5.00^a,b^	101.6 ± 8.1^a,b^
	60		23.25 ± 2.32	201.97 ± 4.91^a^	109.3 ± 9.8^a^
5.0	10	41.43	30.15 ± 3.01	183.82 ± 5.75^a,b^	29.7 ± 4.3^a,b^
	20		31.89 ± 3.12	179.24 ± 6.26^b^	25.1 ± 19.1^b^
	30		31.21 ± 3.12	181.03 ± 5.08^b^	26.9 ± 14.3^b^
	40		26.36 ± 2.36	193.78 ± 5.47^a^	39.7 ± 6.7^a^
	50		26.24 ± 2.62	194.10 ± 5.05^a^	40.0 ± 5.7^a^
	60		27.43 ± 2.74	190.97 ± 8.28^a,b^	36.8 ± 4.5^a,b^
5.5	10	53.06	31.47 ± 3.15	131.79 ± 4.11^b^	41.5 ± 14.9^b^
	20		28.98 ± 2.90	136.58 ± 4.66^b^	46.3 ± 11.6^a^
	30		26.67 ± 2.67	141.02 ± 3.32^a,b^	50.8 ± 0.6^a,b^
	40		21.93 ± 2.19	150.14 ± 3.45^a^	59.9 ± 23.4^b^
	50		31.48 ± 3.15	131.76 ± 2.98^b^	41.5 ± 18.3^a,b^
	60		30.30 ± 3.03	134.04 ± 3.91^b^	43.8 ± 7.5^b^

^a,b,c^The same letters next to values of lead uptake calculated according to the Eq. (1) or Eq. (2) at certain pH value (column) indicate the lack of statistically significant differences between means (p < 0.05), n = 6

**Fig. 1:**
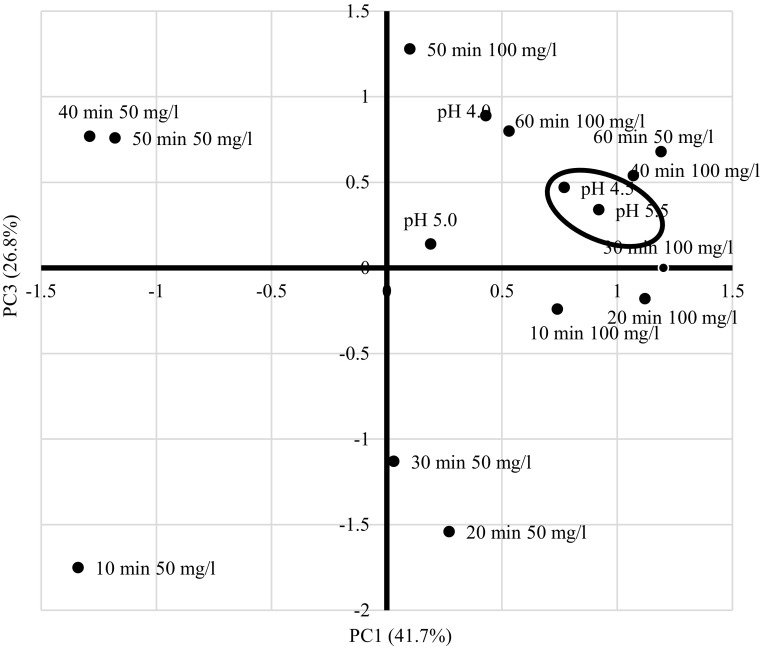
PCA analysis of corrected q_corr_ (lead uptake) obtained at different times and initial lead concentrations (scores) under various pH conditions (loadings) for the first three components; circles indicate correlated loads and scores

Principal Component Analysis of lead(II) uptake was carried out only for results obtained from the Eq. (2). Scripts prepared by Beaumont (2012) were applied for all calculations. Different pH values were considered as loadings and lead uptake values (q_corr_) obtained at different time intervals and two different lead initial concentrations (50 and 100 mg/l) were considered as scores (samples). Correlation matrix (Table 3) showed significant dependencies among considered components (correlation factors above 0.3) that formed two clusters. The p value obtained in Cortest-Bartlett test was relatively low (0.00004), yet it allowed continuing the analysis. In case of both initial lead concentrations it was shown that the first three components explained over 95% variance which indicates that pH 5.5 is not significant for lead biosorption carried out by *A. platensis* so this value could be omitted in further experiments. The strongest correlation (r = 0.8990) was found between pH 4.5 and pH 5.5 (Fig. 1).

**Table 3:** Correlation matrix for lead initial concentrations 50 and 100 mg/l

	pH 4.0	pH 4.5	pH 5.0	pH 5.5
pH 4.0	1.0000			
pH 4.5	0.8055	1.0000		
pH 5.0	0.3671	0.5760	1.0000	
pH 5.5	0.7229	0.8990	0.3605	1.0000

Squares indicate clusters of correlated data

It was also indicated that q_corr_ values obtained after 20, 30 and 40 min contact time are correlated with pH 4.5 when initial metal concentration was 100 mg/l (Fig. 1). Another conclusion is that Pb^2+^ recovery at 100 mg/l is correlated with pH values 4.0, 4.5 and 5.5. Moreover, it was found that shaking time is less significant factor in biosorption phenomenon. The optimum parameters for lead(II) biosorption carried out by *A. platensis* are pH 4.5, initial Pb^2+^ concentration 100 mg/l and shaking time 60 min.

The first principal component was strongly correlated with five of the original variables at lead initial concentration 100 mg/ml: 10, 20, 30, 40 and 60 min (Table 4). This suggests that the lead uptake increases in time of the process. Furthermore, the first principal component correlates most strongly with the time 30 min which suggests that it has got the most significant impact on lead biosorption. It might be also stated that metal biosorption reaches equilibrium after 40 min. The third principal component influences more variables, however, that influence is much weaker since it explains 28.6% of the variance while the first principal component explains 41.7% of the variance. Nevertheless, based on values obtained for the third component it might be stated that the equilibrium of lead(II) biosorption is reached within 30 min at metal initial concentration 50 mg/l. Another conclusion obtained from analysing the third component is that at lead initial concentration 100 mg/l maximum metal uptake was reached after 50 min (the highest principal component value 1.2829, Table 4).

**Table 4:** Correlations between principal components and original variables

Variables	PC1	PC3	PC2
10 min 50 mg/l	− 1.33762	− 1.7467	**2.17395231**
20 min 50 mg/l	0.2700	− 1.5380	− 1.22019895
30 min 50 mg/l	− 0.0280	− 1.1301	− 1.22599243
40 min 50 mg/l	− 1.2922	**0.7610**	− 0.05009083
50 min 50 mg/l	− 1.1751	**0.7645**	− 1.09187203
60 min 50 mg/ml	− 1.1917	**0.6764**	− 0.71562856
10 min 100 mg/l	**0.7411**	− 0.2390	0.24560266
20 min 100 mg/l	**1.1202**	− 0.1837	− 0.12150744
30 min 100 mg/l	**1.1986**	0.0028	− 0.08520182
40 min 100 mg/l	**1.0654**	**0.5373**	**0.58681293**
50 min 100 mg/l	0.0998	**1.2829**	**0.84502001**
60 min 100 mg/ml	**0.5296**	**0.8037**	**0.65910415**

Values marked in bold indicate significant correlations

**Fig. 2:**
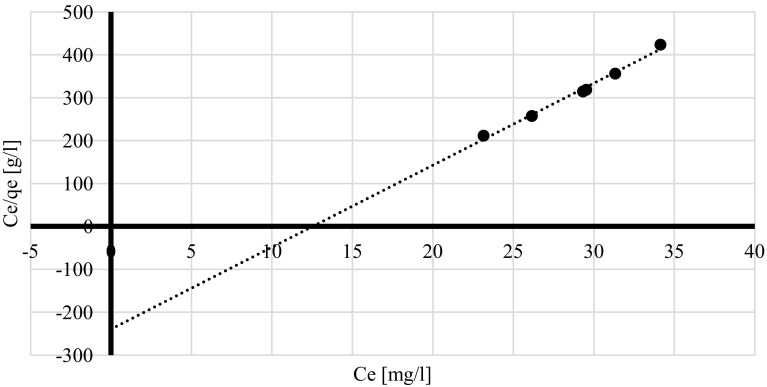
Langmuir isotherm of lead(II) sorption by living cells of *A. platensis* at different time intervals [pH = 4.5, lead(II) initial concentration = 100 mg/l, rotary speed = 200 rpm]

**Fig. 3:**
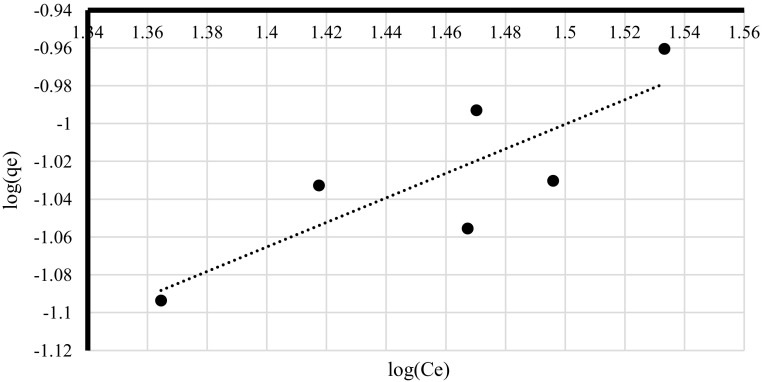
Freundlich isotherm of lead(II) sorption by living cells of *A. platensis* at different time intervals [pH = 4.5, lead(II) initial concentration = 100 mg/l, rotary speed = 200 rpm]

**Fig. 4:**
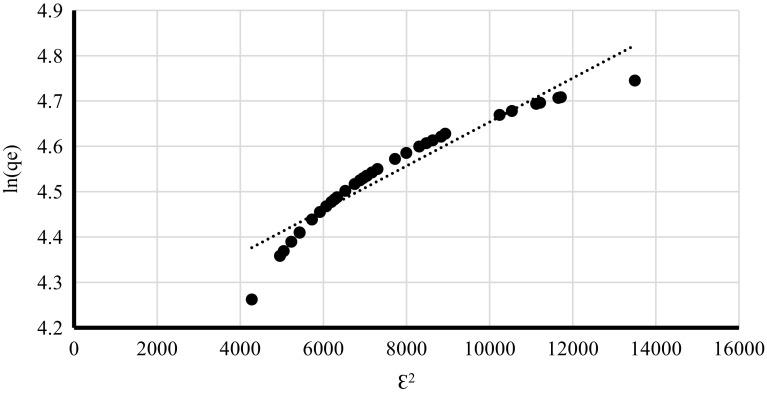
Dubinin–Raduskhevich of lead(II) sorption by living cells of *A. platensis* at different time intervals [pH = 4.5, lead(II) initial concentration = 100 mg/l, rotary speed = 200 rpm]

**Fig. 5:**
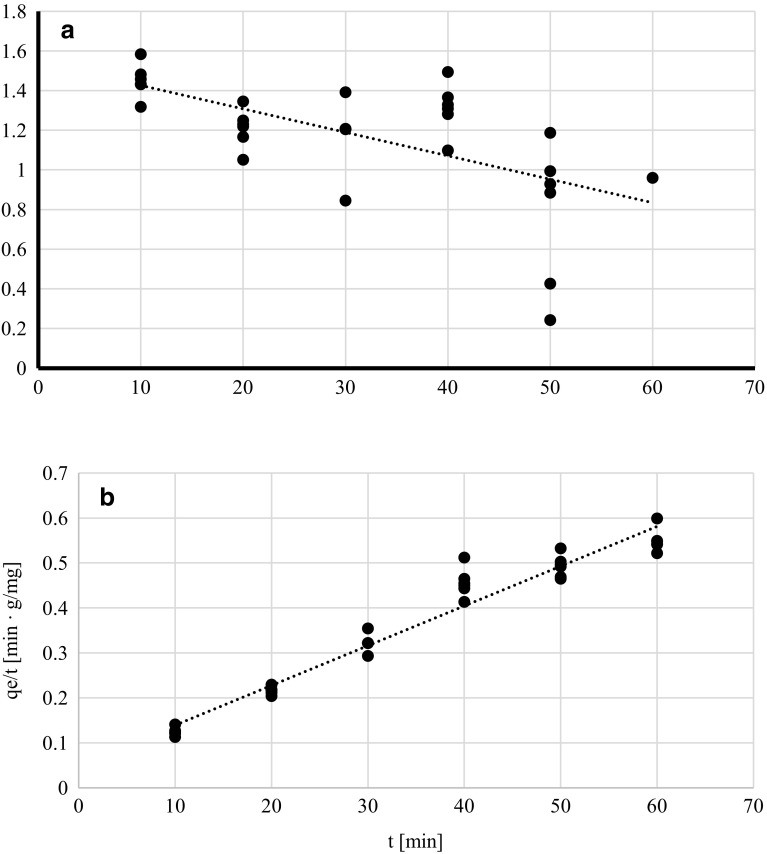
Pseudo-first (**a**) and pseudo-second (**b**) kinetic models of lead(II) sorption by living cells of *A. platensis* at different time intervals [pH = 4.5, lead(II) initial concentration = 100 mg/l, rotary speed = 200 rpm]

Freundlich model verifies if the biosorbent surface is homogenous while Langmuir isotherm allows determining the affinity of metal ions to the biosorbent and estimating the number of binding sites (Volesky 2004). Dubinin–Raduskhevich model is used for estimating material porosity and the apparent energy of adsorption (Hutson and Yang 1997). Freundlich, Lngmuir and Dubinin–Radushkevich isotherms were plotted for q_corr_ obtained at different time intervals at pH 4.5 and lead(II) initial concentration 100 mg/l (Figs. 2, 3, 4) since those conditions were indicated by the PCA as the most optimum for the biosorption process. The experimental data fitted best Langmuir and Dubinin–Radushkevich models (Table 5). In the case of pseudo-first kinetic model it was demonstrated that biosorption performance did not fit the linear model (Fig. 5a) because it was changing its course after the first 20 min of the process—the lead uptake did not increase linearly after that time. This means that the phenomenon considered in the current study did not follow pseudo-first order. On the other, the experimental data fitted pseudo-second kinetic model very well (Fig. 5b).

**Table 5:** The summary of sorption isotherms

Isotherm type	Isotherm constants	Regression coefficient [R^2^]
Langmuir	Q_max_ = 254.4, b = 0.020361	0.9896
Freundlich	K_f_ = 0.010629, n = 1.541545	0.6854
Dubinin–Radushkevich	qs = 64.70 mg/g, K_ad_ = 100 mol^2^/J^2^	0.9124
Pseudo-first order	k_1_ = 1.54/min	0.3755
Pseudo-second order	q_2_ = 0.0512 mg/g, k_2_ = 0.0088 [min·g/mg]	0.9551

## Discussion

Various authors have demonstrated that lead(II) biosorption reaches low efficiencies at pH below 4.0 (Gong et al. 2005b; Wang and Chen 2006; Raoof et al. 2006; Benaïssa and Elouchdi 2007) so in the recent study only the range from 4.0 to 5.5 with 0.5 increment was tested. Hydrogen cations at high concentrations compete for active binding sites with heavy metal ions. On the other hand, lead precipitation occurs at pH above 5.5 which inhibits metal recovery by the biomass (Xuan et al. 2006; Oluyemi et al. 2012; Samra et al. 2014). Based on the obtained results, pH 4.0 and 4.5 provided conditions for more efficient Pb^2+^ recovery which might be explained by the fact that only ionic forms of heavy metals are available to the microbial biomass.

Chen and Pan (2005a) demonstrated that lead did not cause significant damage of *A. platensis* cells over 7 days of incubation at metal concentration ranging up to 20 mg/l. In the current study, higher heavy metal concentration increased chances of “contacting” ions with active binding sites which resulted in enhanced sorption. The adsorption of metal ions by filter paper has not been considered in previous studies where the same procedure of separating biosorbent from test solution was applied (Gong et al. 2005b; Parvathi et al. 2007; Şeker et al. 2008; Solisio et al. 2008). Therefore, it would be needed to revise results and findings reported in cited papers to verify actual efficiencies of lead(II) removal by microbial biomass.

According to the results obtained in PCA the strongest correlation was noted between pH 4.5 and 5.5 (Fig. 1) which might be explained by the fact that at pH 5.5 partial precipitation of lead occurs which means that metal ions are less available to cyanobacteria cells (Akar and Tunali 2006) while the highest metal recovery was obtained at pH 4.5. On the other hand, pH values 4.0 and 4.5 are the most significant parameters in lead(II) biosorption. That correlation could be explained by the fact that at both lead initial concentrations extending the time of shaking experimental flasks did not improve lead recovery, except for experiments carried out at 100 mg of Pb^2+^/L at pH 4.5 where slight improvement of q_corr_ values was noted (Tables 1, 2).

Another strong correlation was noted between pH 4.0 and 4.5 (Fig. 1) and it might be explained by the fact that biosorption was the most effective at these two pH values at lead initial concentrations 50 and 100 mg/l, respectively. Strong correlation also occurred between pH 4.0 and 5.5 (Table 3). Possible explanation to this phenomenon could be that at those pH values lead uptake (q_corr_) was increasing up to 30 or 40 min and it was decreasing afterward. In conclusion, PCA was found useful for analysing biosorption data obtained from preliminary studies.

There was a higher regression coefficient obtained for Langmuir isotherm (Table 5) which indicates that the main mechanism responsible for lead(II) recovery was physical absorption. It might be concluded that heavy metal ions were organised in a monolayer (Volesky 2004) but their affinity to cell walls of tested cyanobacterium was relatively low (Table 5) due to the fact that *b* constant was very low. This might explain why after first 10 min q_corr_ values were high and they decreased after another 10 min. On the other hand, relatively high Q_max_ value (254.4 mg/g of d.m.) indicates that cyanobacterium biomass could be more effective at higher Pb^2+^ concentrations or remove the metal more effectively after further optimisation. The value of that constant also suggests that there are many binding sites available on the surface of the cyanobacterial cell walls. Moreover, results obtained for Freundlich model indicate that the biosorbent surface was not homogenous (Volesky 2004) which is not surprising when complex chemical composition and structure of cell walls are considered (Van Eykelenburg 1978). High material porosity was also confirmed by the data obtained from Dubinin–Raduskhevich model and the value of free sorption energy E, which was higher than 8 (Table 5), confirmed that the biosorption phenomenon performed by living cells of *A. platensis* was based on the chemical sorption (Erhayem et al. 2015). Additionally, the fact that the process follows according to the pseudo-second order confirms that chemical biosorption was very significant mechanism taking place during processes described in the current paper.

The most significant factor influencing lead(II) biosorption by living cells of *A. platensis* is pH and initial metal concentration, while the influence of the contact time is less significant. Moreover, when filter paper is used for separating biomass from the test solution, it has to be determined how much metal is absorbed by that material or that step should be replaced with other separation techniques (i.e. centrifugation). PCA indicated that pH 5.5 was not significant for lead(II) biosorption under tested conditions and optimum parameters for this process were initial metal concentration 100 mg/l, pH 4.5 and contact time 60 min, however, equilibrium was reached after 30 min. It seems that physical sorption is the main mechanism of the described processes. Further studies are necessary to investigate the mechanism of Pb^2+^ biosorption by living cells of *A. platensis* and examine the influence of other factors that have an influence on that phenomenon. Moreover, it should be verified if cyanobacterium cells survive continuous sorption–desorption cycles and how that process could be optimised.
